# Brain activation in older adults during odor identification is related to ApoE, t-tau/Aβ_1–42_, and hippocampal volume

**DOI:** 10.1016/j.neurobiolaging.2025.02.001

**Published:** 2025-02-12

**Authors:** Abigail Albertazzi, Claire Murphy

**Affiliations:** a San Diego State University Department of Psychology, San Diego, CA 92182, USA; b University of California, San Diego Department of Psychiatry, La Jolla, CA 92093, USA

**Keywords:** Olfaction, Biomarker, Genetic risk, fMRI, Structural MRI

## Abstract

Despite altered odor identification preceding and predicting Alzheimer’s disease (AD) cognitive decline, an inadequate understanding of how AD pathology affects odor memory functions limits its use as a preclinical biomarker. Multivariate linear regression was applied to whole-brain blood-oxygen-level-dependent (BOLD) activations during odor identification task (OID) responses in older adults without dementia (*N* = 36, 44.4 % ε4 carriers, *M*_Age_= 76.61). Apolipoprotein-E ε4 allele status, cerebrospinal fluid levels of total-tau to Amyloid-β_1–42_ and MRI-derived hippocampal volume measures were used as predictors. The predictors described significant BOLD variation in regions that are associated with necessary OID functions and affected by AD neurodegeneration during OID responses; moreover, all predictors were associated with significant (*P* < .001) negative BOLD effects in essential task regions during at least one response condition. This evidence suggests significant pathological effects of AD biomarkers on OID-response neural activity in older adults without dementia and should motivate future combined-biomarker investigations of OID functions in preclinical populations.

## 1. Introduction

A review of sensory impairments in Alzheimer’s disease (AD) indicates that olfactory dysfunction, an alteration in one’s ability to smell and process odors, is a promising biomarker of preclinical AD progression (Murphy, 2019; Wesson et al., 2014). The pathophysiological processes of AD are estimated to begin decades before cognitive symptoms onset, and evidence of olfactory dysfunction in aging and AD indicates that such changes may precede cognitive symptoms of AD by several years and signify an increased risk of developing AD (Albers et al., 2015; Murphy, 2019). Identifying AD development in older adults who have yet to demonstrate symptoms of cognitive decline is important to defining preclinical AD manifestations and implementing treatment interventions. Studies have suggested that combining early AD biomarkers, including olfactory psychophysical measures, increases precision and may strongly predict conversion from mild cognitive impairment (MCI) to AD (Devanand et al., 2008; Heister et al., 2011; Wheeler and Murphy, 2021). Further exploration of the relationships between the appearance of AD pathology and olfactory function in humans, especially neuroimaging investigations of olfactory impairments and AD risk factors during preclinical AD stages, may elucidate manifestations of AD that precede cognitive memory impairments and the validity of olfactory dysfunction as a preclinical biomarker (Albers et al., 2015).

Essential olfactory processing brain regions (i.e., entorhinal and transentorhinal cortices, anterior olfactory nucleus, and olfactory bulb) are notably involved in AD tau pathology prior to the hippocampus and temporal cortex (Braak and Braak, 1991, 1992, 1994, 1995, 1997). Therefore, the initial and heaviest involved regions of AD-tau pathology—essential for emotional regulation and memory functions (including odor memory)—are regions critical to olfactory function (Price et al., 1991; Zhu et al., 2019). Older adults generally demonstrate impaired olfactory information processing—shown as poor olfactory task performance (e.g., odor identification or recognition memory; Albers et al., 2015; Murphy, 2019; Murphy et al., 2002) and reduced blood-oxygen-level-dependent (BOLD) activation of olfactory processing regions and MTL areas—that worsens with increased age (>65years; Murphy, 2019; Bothwell et al., 2023; Cerf-Ducastel and Murphy, 2009). Over the last few decades, several studies have found odor memory to be associated with factors of AD severity in older adults with confirmed AD, MCI, and normal cognition (Gilbert and Murphy, 2004a; Kjelvik et al., 2014; Murphy, 2019; Murphy et al., 2009; Vasavada et al., 2017; Waldton, 1974; Wang et al., 2010). Specifically, impaired odor identification (OID) at baseline has been shown to predict cognitive decline, after 5 years (Albers et al., 2015; Dintica et al., 2019; Schubert et al., 2008), and more accurately than hearing or vision impairment (Fischer et al., 2016; Murphy, 2019), and above and beyond factors of age, sex, education, apolipoprotein-E (APOE) epsilon 4 (ε4) allele status, and episodic memory function (Wilson et al., 2007).

APOE allele expression, cerebrospinal fluid (CSF) Aβ and tau levels, and MRI-derived reduced hippocampal volume (HV) or hippocampal occupancy measures are often used to classify genetic, pathological severity, and neurodegenerative risk status, respectively (Bondi et al., 2005; Brewer et al., 2009; Corder et al., 1993; Galasko & Shaw, 2017; Haase et al., 2013; Heister et al., 2011; Huang, 2010; Kim et al., 2009; Mahley et al., 2006; Ochs et al., 2015; Sperling et al., 2014; Wheeler and Murphy, 2021). Although their specific influences on AD development remain unclear AD these biomarkers have all been individually associated with cognitive decline, pathological severity, and impaired OID performance in older adults with AD, MCI, and normal cognition (Albers et al., 2015; Blennow and Zetterberg, 2018; Devanand et al., 2008; Fischer et al., 2016; Graves et al., 1999; Gilbert and Murphy, 2004a, 2004b; Jack et al., 1992; Josefsson et al., 2017; Kjelvik et al., 2014; Lafaille-Magnan et al., 2017; Murphy, 2019; Murphy et al., 1998, 2003; Nelson et al., 2012; Oleson and Murphy, 2015; Olofsson et al., 2010; Weiner et al., 2017; Wilson et al., 2007).

Several recent studies have reported significant associations of blood as well as PET imaging markers with odor identification, some involving longitudinal data (Tu et al., 2020; Tian et al., 2023; Bothwell et al., 2023), suggesting that odor identification may be related to developing neuropathology. Murphy, Jernigan & Notestine (2023)reported that left hippocampal volume was associated with odor identification performance in Alzheimer’s patients. Recent studies have demonstrated an association between odor identification and significantly reduced brain volumes in older adults (Bothwell et al., 2023; Tian et al., 2023). Beyond structural studies, investigating brain activation with functional MRI during olfactory tasks such as odor identification is particularly important because it is capable of demonstrating changes in brain function. In particular, functional studies are critical to investigate whether the brain can compensate for decrements in structural integrity. Investigating brain response separately during both correct and incorrect responses would allow for addressing potential differences in their underlying odor identification networks, particularly whether compensation is required for correct odor identification.

Combining biomarkers in a multivariate approach is proposed to best describe AD development in preclinical populations; therefore, the present study investigates the relationships between AD biomarkers and BOLD activity during OID-task responses in older adults without dementia using multivariate linear regression. It is hypothesized that APOE-ε4 status, CSF-tau burden, and estimated HV biomarkers will significantly affect BOLD activity in regions associated with OID functions; and that the effects of the biomarkers on BOLD responses will reflect AD-related decline (i.e., greater risk being associated with negative neurofunctional effects).

## 2. Materials and methods

### 2.1. Participants & measures

Participants were 36 older adults without dementia (*M*_Age_ = 76.61; SD = 4.21; *N*_*Female*_ = 20) recruited from the UCSD Alzheimer’s Disease Research Center (ADRC) (See Supplementary Table 1). As volunteer research participants at the UCSD ADRC, they underwent comprehensive assessment that includes genetic APOE allele marking, CSF lumbar puncture, and yearly clinical assessments (See Edmonds et al., 2021). Inclusionary criteria for the study required participants to be above the age of 65, clinically nondemented with no signs of alternative neurological insult or neurodegenerative disease that could affect cognitive and sensory functions. A diagnosis of cognitively normal was determined at each annual visit by the consensus of a multidisciplinary team (2 senior neurologists and a neuropsychologist). 16 were APOE-ε4 carriers, 20 were noncarriers, and four (two female) had MCI.

CSF lumber puncture was performed in accordance with recommended best practices (See eMethods, available from Dryad [doi.org/10.6076/D1F300]). CSF Amyloid β and tau levels were analyzed by the UCSD ADRC using the Lumipulse G^®^ assay. CSF total-tau (t-tau) and Amyloid β_1–42_ levels were used as a ratio (t-tau/Aβ_1_–_42_) to reflect tau burden for each participant (range = 0.171–2.9, *M* = 0.521, SD = 0.5205; Edmonds et al., 2021).

HV was investigated as two separate measurements: a measure of hippocampal volume in the right hemisphere that controls for individual intracranial volume (ICV) and the Avg-HOC measure that accounts for inferior lateral ventricle volume, age, and biological sex. Both measures are derived from raw structural MRI volumes and are used to reflect hippocampal integrity in clinical studies. T1-weighted images were processed using standard automated procedures from the FreeSurfer image analysis suite available online (Version 7.2.0 http://surfer.nmr.mgh.havard.edu) to obtain hippocampal and intracranial volume estimates for all participants. The measure of right hemisphere HV used in analyses represents the ratio of raw HV to estimated intracranial volume (ICV; right HV/ICV). Conversely, the measure of Avg-HOC, provided by the UCSD ADRC, represents an age- and sex-normative score of the ratio of bilateral hippocampal volume to the sum of bilateral hippocampal and inferior lateral ventricle volumes. Both mathematical adjustments to the HV variables are used to account for congenital differences in hippocampal volume.

#### 2.1.1. BOLD activity during odor identification-task responses

An event-related fMRI design was used to measure BOLD whole-brain activations during correct and incorrect OID task responses. The scan protocol included two separate runs of the OID task. Odor stimuli were delivered one at a time to participants in the scanner using a computer-operated olfactometer. Participants were instructed to smell the presented odor stimulus and identify it by selecting (via button-box) which of the four verbal odor labels presented on a screen best matched the odor they smelled. MATLAB controlled stimulus presentation, screen presentation of verbal odor labels, and recorded responses in synchrony with olfactometer odor stimulus delivery. OID performance was defined as the number of correct OID responses, and BOLD activity during correct and incorrect OID responses were extracted.

### 2.2. Magnetic resonance imaging acquisition and preprocessing

MRI was conducted at the UCSD Center for Functional MRI (CFMRI) using a 3 T General Electric MR750 Scanner. Functional images were collected using a standard gradient echo EPI pulse sequence to acquire T2 * -weighted functional images (imaging matrix: 64×64, 36 axial slices, field of view = 19.2 cm, repetition time = 2 s, echo time = 30 ms, flip angle = 90°). Structural T1-weighted images were also acquired for each participant using whole-brain fast spoiled gradient echo MRI sequence (field of view = 24 cm, Locs per slab = 176, resolution =.94x.94×1.2 mm3, repetition time = 8.2 ms, echo time = 3.18 ms, flip angle = 8°).

Functional data were minimally preprocessed using fMRIPrep v21.0.01 (Esteban et al., 2018), with boundary-based registration and FreeSurfer surface preprocessing disabled, and then smoothed with an 8-mm Guassian kernel full-width half maximum (FWHM). The Analysis of Functional NeuroImage (AFNI; Cox, 1996) open-source software was used to process each participant’s concatenated runs based on the specified task response condition (e.g., activation during correct and incorrect responses) with 3dDeconvolve (Ward, 2002). The 3dDeconvolve output contains fit coefficients (i.e., beta weights) for each voxel, indicating the amplitude of the signal model for each contrast and corresponding t-statistics. An individual voxel *P* value of .005 and a minimum cluster size of 30 voxels was determined a priori to best estimate significant relationships between BOLD brain activity during correct and incorrect OID responses and three variables of interest (APOE-ε4 status, HV, and CSF t-tau/Aβ_1–42_ levels).

### 2.3. Statistical analyses

#### 2.3.1. Olfactory network GLM analyses

To identify the network of regions activated during OID, we conducted GLM analysis on activation during correct odor identification in the cognitively normal older adults without genetic risk for AD, the non-carriers of the ε4 allele. A one sample *t*-test was used to detect the underlying olfactory network. The groups level map was then thresholded at a voxel level of .005 and a cluster threshold of 30 voxels to control for false positive activation clusters.

We also investigated the regions activated during correct vs incorrect OID overall; i.e., a contrast analysis was performed to identify differences in task activation between correct and incorrect identifications (correct > incorrect identifications). We were particularly interested in areas that are activated during correct but not incorrect identifications in order to detect areas that might be involved in compensation. The difference in activation estimates between correct and incorrect identifications was tested using a one-sample *t*-test to detect the olfactory network underlying correct identification of odorants. The groups level map was then thresholded at a voxel level of .005 and a cluster threshold of 30 voxels to control for false positive activation clusters.

#### 2.3.2. Multivariate linear regressions with AD risk factors and olfactory BOLD as outcome

Multivariate linear regression was used to investigate the relationship between AD risk factors and olfactory functioning underlying OID (i.e., BOLD activity during olfactory task responses) in older adults without dementia. The predictors and covariates used in the regression model were, as follows: APOE-ε4 status coded as ε4(−) = 0 vs ε4(+) = 1, CSF ratio centered at 0.5, and a measurement of HV (Model 1: right HV/ICV or Model 2: Avg-HOC) as predictors; sex, age centered at 75, and two interaction terms ε4 status*sex and sex*age, determined a priori, as relevant covariates. The two HV measures were analyzed separately, resulting in two different models (Model 1: right HV/ICV; Model 2: Avg-HOC). We found an R^2^ of .91 for the relationship between DRS scores and functional activation in the right parahippocampal gyrus during odor recognition memory in previous research (Kapoulea and Murphy, 2020), and we sought to limit the number of variables in the current analyses; thus, right HV and Avg-HOC measures were analyzed here. 4 participants were included in the first model that did not have Avg-HOC and were excluded from the second model, but variable distributions and means did not significantly differ. Both regression models were fit to the imaging data from correct and incorrect OID responses and BOLD clusters that were found to be significantly associated with the predictor variables and both models were reported. Additionally, the mean threshold of BOLD activity for each reported cluster was extracted and used to reveal model fit (adjusted R^2^) as well as significant unique effects of the model variables.

## 3. Results

### 3.1. Olfactory network GLM analyses

GLM identified four clusters of significant activation when ε4 non-carriers correctly identified odors (Hits). Activation was found in a large cluster (k = 22358 voxels) including the piriform cortex, entorhinal cortex, parahippocampal gyrus, hippocampus, precuneus, inferior frontal gyrus, middle frontal gyrus, precentral gyrus, postcentral gyrus, basal ganglia, inferior parietal lobule, superior parietal lobule, angular gyrus, thalamus, and insula. Two smaller clusters of positive activation during hits were detected in the right lingual gyrus (k = 31 voxels) and left posterior cingulate cortex (k = 31 voxels). A single cluster of negative activation during hits was detected in the left parietal white matter (k = 117 voxels). A full description of regions significantly associated with correctly identifying odors can be found in Table 1.

Five significantly different clusters of activation were found to be related to the contrast of correct identification to incorrect identification. All clusters showed a negative directionality, suggesting that lesser activation was related to correct identifications relative to incorrect identifications of odorants. The largest cluster (k = 8240 voxels) covered large portions of the frontal and parietal lobes, including the bilateral MFG, IFG, SFG, precentral gyrus, postcentral gyrus, middle cingulate gyrus, precuneus, inferior parietal lobule, superior parietal lobule, supramarginal gyrus, and posterior cingulate gyrus. 4 smaller clusters of negative activation were found, including a cluster including the bilateral caudate and right insula (k = 69 voxels), a cluster including portions of the bilateral lingual gyrus, left calcarine gyrus, and cerebellum (k = 38 voxels), a cluster including portions of the right pallidum and thalamus (k = 31 voxels), and a cluster including portions of the right superior parietal lobule, inferior parietal lobule, and angular gyrus (k = 31 voxels) Table 2.

### 3.2. Multivariate linear regression of fMRI responses

APOE-ε4 status, HV, and CSF t-tau/Aβ_1–42_ levels were all found to negatively affect BOLD activity in the older adults during correct and/or incorrect responses. Regression beta-weight coefficients for the clusters of BOLD activity significantly associated with the APOE-ε4 status predictor revealed a negative main effect for APOE-ε4 carriers during both correct and incorrect OID responses. HV predictors were associated with significant negative main effects on BOLD brain activation during correct OID responses; such that, HV below the sample average was associated with hyperactivation of the affected regions.

The CSF predictor had no significant main effects during correct OID responses; similarly, HV had no significant main effects during incorrect OID responses. CSF t-tau/Aβ_1–42_ levels above 0.5 were associated with less BOLD brain activity during incorrect OID responses Table 3.

### 3.3. Odor identification-correct responses

#### 3.3.1. ε4 status

**Fig. 1.**
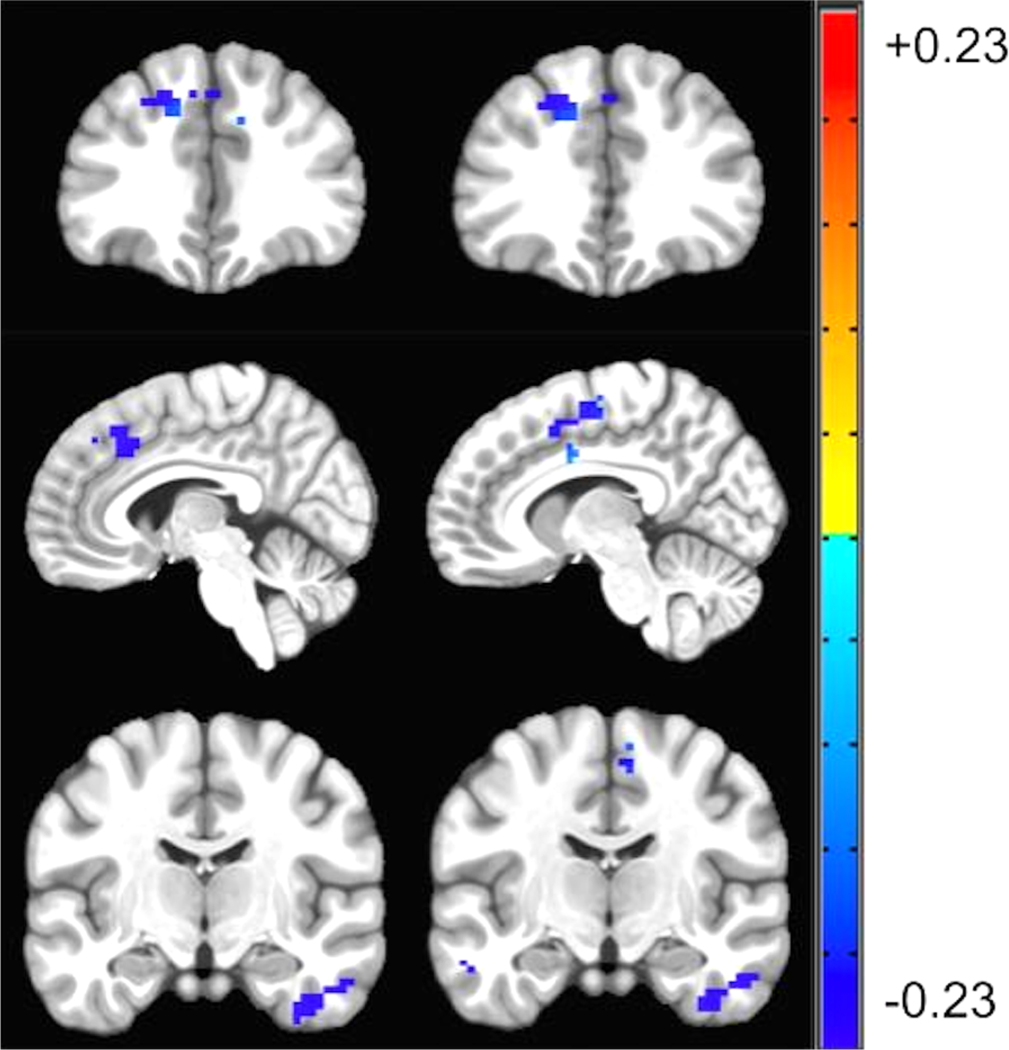
Clusters of BOLD activity that were negatively associated with APOE-ε4 status during correct OID responses. Clusters revealed by Model 1 are shown on the left and Model 2 on the right. (Top) Model 1 (164 voxels) and Model 2 (277 voxels) included the L. superior frontal gyrus, L. superior medial gyrus, L. middle frontal gyrus, L. SMA, R. superior medial gyrus, bilateral anterior cingulate cortices, and bilateral middle cingulate cortices. (Middle) Model 1 (48 voxels) and Model 2 (45 voxels) included the R middle cingulate cortex, R. superior medial gyrus, R. SMA, L. superior medial gyrus, R. anterior cingulate cortex, and L. SMA. (Bottom) Model 1 (55 voxels) and Model 2 (56 voxels) included the R. ERC, R. fusiform gyrus, R. parahippocampal gyrus, and R. inferior temporal gyrus.

In both models, being APOE-ε4 + was generally associated with less BOLD activity in three specific clusters during correct OID responses. These clusters appeared in the left (Figure 1. Top) and right (Figure 1. Middle) SFL and right MTL (Figure 1. Bottom).

Both models had similar adjusted R^2^ values for the left (Adj R^2^_Model 1_ = 0.337 & Adj R^2^_Model 2_ = 0.382) and right (Adj R^2^_Model 1_ = 0.442 & Adj R^2^_Model 2_ = 0.486) SFL clusters of BOLD activity, and, despite the difference in HV measurement used by each model, the unique effect of APOE-ε4 status remained stable for both clusters (Left *β*_*Model 1*_ = −0.22; vs *β*_*Model 2*_ = −0.23; Right *β*_*Model 1*_ = −0.32, *β*_*Model 2*_ = −0.33). Some subtle differences in covariate unique effects were present across the models—which was expected due to the complex relationship between age, sex, and HV—however, both models found the ε4xSex interaction term to have a significant positive effect on the mean BOLD threshold at the right and left SFL clusters.

The mean threshold of BOLD activity in the right MTL cluster was predicted similarly by both models (Adj R^2^_Model 1_ = 0.369; Adj R^2^_Model 2_ = 0.406). Significant unique contributions of APOE-ε4 status and the covariates of age, ε4xSex, and AgexSex on the cluster of right MTL BOLD activity were also present in both models at similar thresholds.

#### 3.3.2. CSF - correct odor identification

CSF t-tau/Aβ_1–42_ levels were not found to significantly affect BOLD activity during correct OID responses by either model at the .005 level.

#### 3.3.3. Hippocampal volume - correct odor identification

Both HV measures were associated with hyperactivation at the bilateral cuneus and precuneus during correct OID responses, in Model 1 (64 voxels) and Model 2 (63 voxels); such that, decreased HV was associated with hyperactivation. Model 2 appeared to predict bilateral cuneus and precuneus activity slightly better (Adj R^2^ = 0.365) than Model 1 (Adj R^2^ = 0.299). No other predictors nor covariates significantly correlated with the cluster in Model 1; however, Model 2 found age to have a small negative correlation (*β* = −0.02; *P* = 0.006) with cuneus and precuneus activity.

**Fig. 2.**
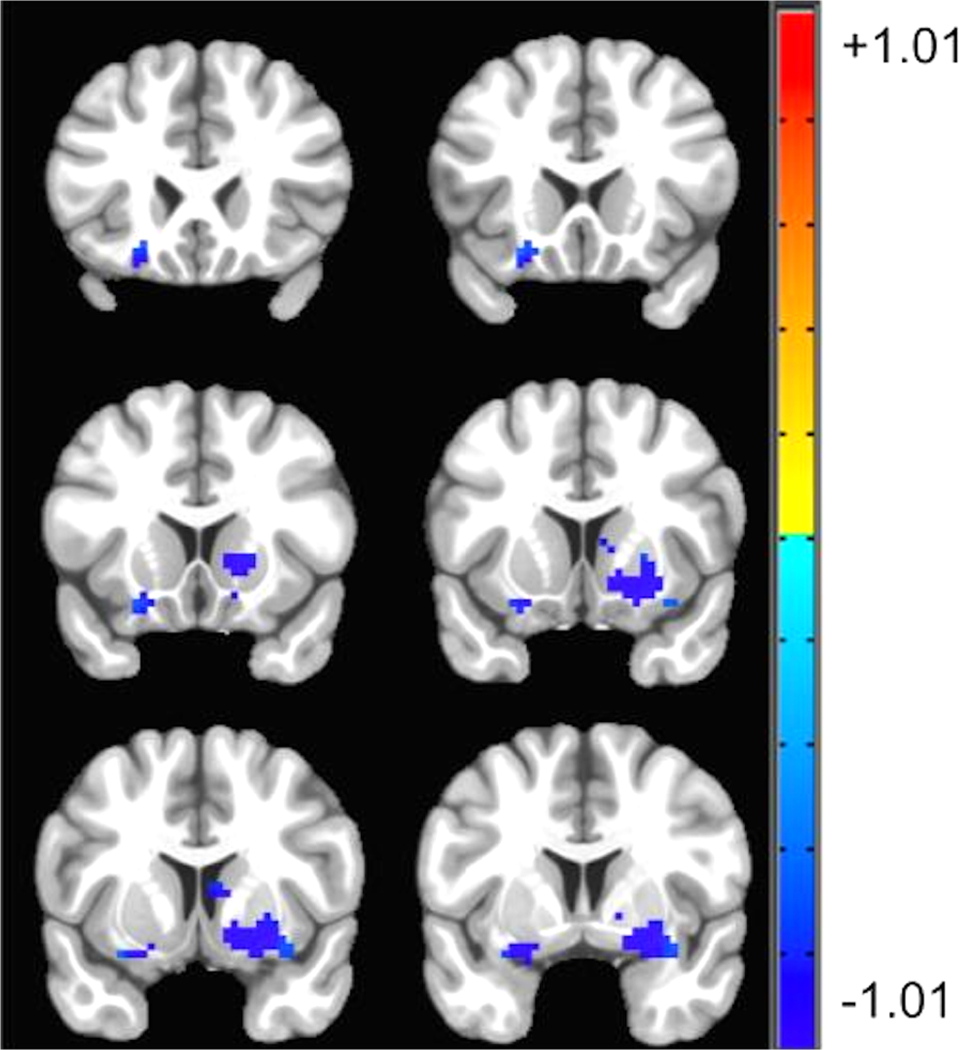
Two clusters of BOLD activity negatively associated with Avg-HOC during correct OID responses, in Model 2. The right hemisphere cluster (171 voxels) included the piriform cortex, posterior OFC complex, putamen, caudate nucleus, pallidum, hippocampus, amygdala, insula, and olfactory cortex; the left hemisphere cluster (74 voxels) included the piriform cortex, anterior agranular insula complex, posterior OFC complex, inferior frontal gyrus (orbitalis), insula lobe, olfactory cortex, amygdala, putamen, pallidum, superior orbital gyrus, inferior frontal gyrus, rectal gyrus, and temporal pole.

Two additional clusters of hyperactivation during correct OID responses were associated with HV (specifically, Avg-HOC) in Model 2 (Figure 2), showing added value of Avg-HOC as a measure of HV when investigating olfactory dysfunction in older adults without dementia. The clusters were located at right and left primary olfactory processing regions (i.e., piriform cortex, posterior OFC complex, hippocampus, amygdala) and were relatively large in size.

The model was a better fit to the cluster of olfactory regions in the right hemisphere (Adj R^2^ = 0.470; *P* = 0.002) than the left hemisphere cluster (Adj R^2^ = 0.261; *P* = 0.045). Avg-HOC and CSF t-tau/Aβ_1–42_ levels were significantly associated with activity in the right primary olfactory regions and MTL, as well as age and the interaction of AgexSex; Avg-HOC was the only significant variable associated with BOLD activity in the left olfactory cortex and MTL. The unique effects of Avg-HOC were relatively large for both clusters, though larger for the left (*β* = −3.01; *P* < 0.001) than the right hemisphere cluster (*β* = −1.20; *P* < 0.001).

#### 3.3.4. Odor identification-incorrect responses

##### 3.3.4.1. ε4 status.

**Fig. 3.**
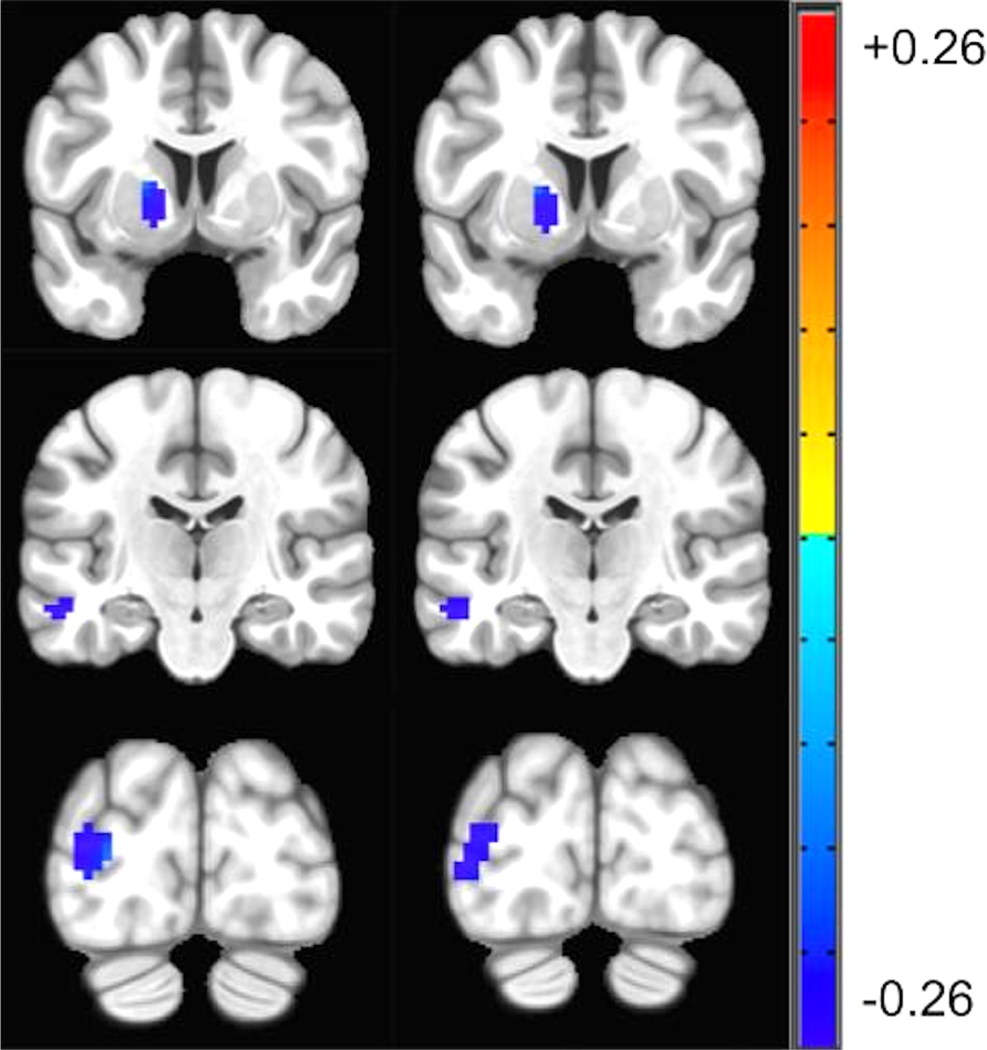
Clusters of BOLD activity that were negatively associated with APOE-ε4 status during incorrect OID responses. Clusters revealed by Model 1 are shown on the left and Model 2 on the right. (Top) Model 1 (52 voxels) and Model 2 (56 voxels) included the L. putamen, L. pallidum, and L. caudate nucleus. (Middle) Model 1 (44 voxels) and Model 2 (59 voxels) were located at the L. middle occipital gyrus. (Bottom) Model 1 (31 voxels) and Model 2 (51 voxels) included the L. middle and L. inferior temporal gyri.

In both models, being APOE-ε4 + was associated with less activity in 3 clusters in the left hemisphere, including the corpus striatum (Figure 3. Top), middle occipital gyrus (Figure 3. Middle) and MTL (Figure 3. Bottom). Activity in the left corpus striatum was better described by Model 2 (Adj R^2^ = 0.516; *P* = < 0.001) than by Model 1 (Adj R^2^ = 0.3659; *P* = 0.0045). The unique effects of APOE-ε4 status were similar in both models (*β*_*Model 1*_ = −0.244, *P* < 0.001; *β*_*Model 2*_ = −0.249, *P* < 0.001). Е4 allele status was the only variable found to be significantly associated with variation in the left corpus striatum BOLD signal in Model 2 (See Table 4). Model 1 found CSF t-tau/Aβ_1–42_ levels (*β* = −0.116; *P* < 0.001), and significant covariate associations with age and the ε4xSex interaction, to be associated with the level of activity in the cluster.

The activation in the left middle occipital gyrus associated with ε4 status during incorrect OID responses was better described by Model 2 (59 voxels; Adj R^2^ = 0.454; *P* = 0.003) than Model 1 (44 voxels; Adj R^2^ = 0.390; *P* = 0.003). Both models found similar unique associations of ε4 status (*β*_*Model 1*_ = −0.22, *P* < 0.001; *β*_*Model 2*_ = −0.26; *P* < 0.001) and the interaction of ε4xSex (*β*_*Model 1*_ = 0.16, *P* = 0.031; *β*_*Model 2*_ = 0.20, *P* = 0.032). Model 2 revealed an additional significant unique association of the CSF ratio predictor with the level of activity in the cluster (*β* = −0.11; *P* = 0.010), in addition to significant covariate associations with age, sex, and the AgexSex interaction (Table 4).

Activation at the left MTG & ITG, associated with ε4 status during incorrect OID responses, was better described by Model 2 (Adj R^2^ = 0.409; *P* = 0.006) and associated with a stronger negative effect of ε4 status (*β* = −0.41; *P* < 0.001) than in Model 1 (Adj R^2^ = 0.307, *P* = 0.013; *β* = −0.38, *P* < 0.001). All covariables were found to significantly explain the variance of left MTL BOLD activity in Model 1; nearly identical unique covariable associations were revealed in Model 2 apart from sex, which was not found to be significant by the model.

##### 3.3.4.2. CSF ratio t-tau/Aβ_1–42_ - incorrect odor identification.

**Fig. 4.**
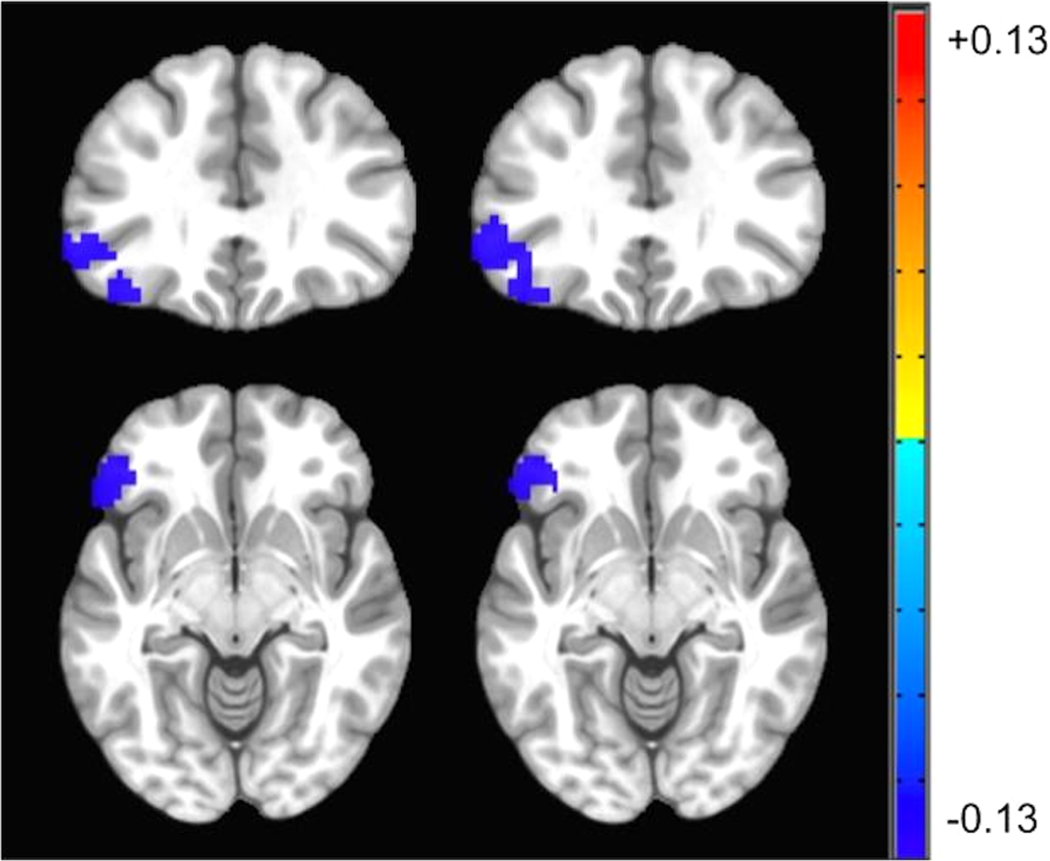
A cluster of BOLD activity at the left OFC found to be negatively associated with CSF t-tau/Aβ_1–42_ levels during incorrect OID responses, found in Model 1 (154 voxels) and Model 2 (166). The cluster included the L. inferior frontal gyrus (orbitalis and triangularis) and L. temporal pole.

CSF t-tau/Aβ_1–42_ ratios above 0.5 were associated with less activation in the left hemisphere OFC and temporal pole during incorrect OID responses (Figure 4).

Both models similarly described the variation in BOLD activity of the cluster (Adj R^2^_Model 1_ = 0.553, *P* < 0.001; Adj R^2^_Model 2_ = 0.533; *P* < 0.001) and revealed similar significant unique effects of CSF t-tau/Aβ levels (*β*_*Model 1*_ = −0.21, *P* = 0.010; *β*_*Model 2*_ = −0.20, *P* = 0.043) and APOE-ε4 status (*β* = −0.18, *P* = 0.009; *β* = −0.16, *P* = 0.029). All covariables were also represented similarly in the models apart from the ε4xSex interaction, which was not found to be significant in Model 2 (Table 5).

##### 3.3.4.3. Hippocampal volume - incorrect odor identification.

HV was not shown to be significantly associated with clusters of BOLD activity (≤ 30 voxels) during incorrect OID responses by either model at the .005 level.

## 4. Discussion

GLM analyses suggested that a range of frontal, parietal, and subcortical structures are involved in the successful identification of odorants, including regions such as the piriform cortex, entorhinal cortex, parahippocampal gyrus, hippocampus, precuneus, MFG, IFG, posterior cingulate, insula, thalamus, caudate, inferior parietal lobule. This is similar to previous analyses of functional connectivity of odor identification networks using independent component analysis (ICA; Frank, Albertazzi and Murphy, 2024) and suggests that coordination of a diverse range of networks involving frontal, parietal, subcortical, temporal, and cerebellar processing regions underlies odor identification.

The observed unique associations of the investigated AD risk factors with BOLD activity during OID responses offer support to pre-existing theories of the relationship(s) between the individual model variables and the development of AD (Albers et al., 2015; Blennow and Zetterberg, 2018; Devanand et al., 2008; Frank et al., 2024; Graves et al., 1999; Heister et al., 2011; Lafaille-Magnan et al., 2017; Murphy, 2019; Murphy et al., 2024; Wesson et al., 2014; Wheeler and Murphy, 2021) and valuable new insight into potential pathological progression in preclinical older adult populations. The risk factors were associated with BOLD activity often attributed to neurodegeneration and cognitive decline (Vasavada et al., 2017; Wang et al., 2010; Kapoulea and Murphy, 2020); generally less BOLD activation with evidence of compensatory effort (hyperactivation; Bondi et al., 2005; Kapoulea and Murphy, 2020). The ε4 status and increased CSF ratios associated with less activity in regions associated with functions necessary for odor identification (odor stimulus processing, recollection memory of an odor, semantic memory of verbal odor labels, inhibitory control, motor control, and other task-critical cognitive functions; (Kjelvik et al., 2012; Price et al., 1991) suggests that poor OID is associated with developing pathology. The greater activation associated with less HV (Right HV/ICV or Avg-HOC) has been previously suggested to reflect greater compensatory effort due to neurological compromise in AD, which may partially explain the observation of this increase in activation specific to correct responses (Bondi et al., 2005; Kapoulea and Murphy, 2020). The additional finding of less Avg-HOC associated with activation in primary olfactory processing regions reflects the complex relationship between hippocampal integrity and olfactory dysfunction in the presence of AD risk and supports future use of Avg-HOC to measure hippocampal atrophy. Further, brain regions associated with each variable indicate the regions associated with OID responses in older adults without dementia and their potential involvement in the development of AD.

### 4.1. APOE-ε4 allele status during odor identification responses

Activation in bilateral SFL and MTL regions and the left corpus striatum associated with ε4 status is related to cognitive function during task performance. Regions of the SFL (i.e., superior frontal gyri and SMA) and corpus striatum are critically associated with motor cognitive functions. The superior frontal gyrus (SFG)—shown to be a key component of the working memory network and mediation of inhibitory control and motor urgency counteracting processes (du du Boisgueheneuc et al., 2006; Hu et al., 2016; Marek and Dosenbach, 2018)—and supplementary motor areas (SMA) are considered critical to the development of intention-to-act and the specification and elaboration of the action (Koyama et al., 2017). The loss of dopaminergic neurons in the olfactory bulb and associated mitochondrial dysfunction has been proposed as one of the underlying mechanisms for changes in brain activation (Tian et al., 2023). Activation of the SFL regions and middle cingulate cortex (MCC) is considered necessary for task performance (Marek and Dosenbach, 2018; Oane et al., 2020). Similarly, activity in the corpus striatum—including the caudate nucleus, putamen, and globus pallidum—is believed to be related to motor and cognitive functions needed for task completion, as these regions are associated with control of conscious and proprioceptive movements (Balleine et al., 2007).

The hyperactivation in the SFL during correct responses and the left corpus striatum during incorrect responses associated with the ε4xSex interaction may be related to underlying response-specific pathological relationships. Separately, APOE-ε4 status and sex are notably associated with differences in olfactory dysfunction in adults with normal cognition, MCI, and AD (Bondi et al., 2005; Filippini et al., 2009; Gilbert and Murphy, 2004a, 2004b; Green et al., 2023; Haase et al., 2013; Kapoulea and Murphy, 2020; Murphy, 2019); furthermore, the ε4xSex interaction has been significantly associated with the rate of AD pathology progression and olfactory dysfunction (Graves et al., 1999; Josefsson et al., 2017; Murphy, 2019; Murphy et al., 2009).

The reduced activity in MTL regions (i.e., EC, hippocampus, middle and inferior temporal gyri) during correct (R MTL) and incorrect (L MTL) OID responses highlights the vulnerability of higher-level olfactory processes in older adults at genetic risk for AD, as these regions are essential for odor memory (Cleland and Linster, 2019; Royet et al., 1999; Royet and Plailly, 2004; Savic, 2002). The EC receives primary olfactory projections (Price et al., 1991; Weismann et al., 2001) and is a major source of afferent input to the hippocampus (Royet and Plailly, 2004; Van Hoesen and Pandya, 1975). Odor information travels to the EC and is then sent to secondary sensory processing regions. Bilateral MTL activation is associated with OID as it is a memory function that requires both recollection (nonverbal) memory of an odor stimulus—a function associated with the right MTL activity—and memory of semantic (verbal) labels to identify the odor—associated with left MTL involvement (Kjelvik et al., 2012; Murphy, 2019; Royet et al., 1999; Royet and Plailly, 2004; Savage, 2002). The findings may, therefore, be related to impairment of the lateralized odor memory functions associated with either hemisphere in older adults genetically at risk for AD. The reduced MTL BOLD activity associated with positive APOE-ε4 status and age over 75 years, as well as the ε4xSex interaction, are congruent with their status as risk factors of AD (Graves et al., 1999; Jack et al., 1992; Josefsson et al., 2017; Kim et al., 2009; Murphy, 2019; Murphy et al., 2009; Wang et al., 2010; Weiner et al., 2017). Older adults who are above the age of 75 and/or ε4 carriers are among those with the highest risk for developing AD (Corder et al., 1993).

### 4.2. CSF Ratio of Total Tau to Aβ_1–42_ During Odor Identification

#### Responses

The reduced activation during incorrect OID responses associated with both APOE-ε4 carrier status and CSF ratios above 0.5 in the left OFC —an area strongly associated with odor memory functions — (Cleland and Linster, 2019; Filippini et al., 2009; Gottfried and Zald, 2005; Price, 2007; Royet et al., 1999; Royet and Plailly, 2004; Sakikawa et al., 2023; Savage, 2002; Savic, 2002; Schoenbaum and Eichenbaum, 1995; Suen et al., 2021; Watanabe et al., 2018) is related to a significant progression of AD-tau pathology (Braak and Braak, 1995; Nojima et al., 2022; Weiner et al., 2017). The OFC —located superior to the olfactory bulbs and temporal poles—is considered critical to sensory integration, learning associations, and controlling and correcting reward-related behaviors (Gottfried and Zald, 2005; Price, 2007; Sakikawa et al., 2023). More importantly, it is considered a principal neocortical element of the olfactory system with a pivotal associative role in olfactory information processing—including recognition, naming, and emotional valency judgment of odor stimuli (Gottfried and Zald, 2005; Schoenbaum and Eichenbaum, 1995). The OFC has extensive connections to all primary olfactory regions, as well as the temporal lobe regions, and has been closely associated with OID odor information processing and the object recognition pathway (Price, 2007; Savage, 2002; Zhou et al., 2019). AD-related tau pathology initially accumulates in transentorhinal cortices and the MTL before involving frontal and parietal regions (Braak and Braak, 1995); suggesting that, despite the OFC not being earliest involved in tau pathology, OFC functions may be affected by the compromise of its connected regions (Zhou et al., 2019).

### 4.3. Hippocampal Volume During Odor Identification Responses

The cuneus and precuneus hyperactivity during correct OID responses associated with reduced HV is consonant with the increased activation of the precuneus commonly found in individuals with AD, MCI, or at risk for AD (e.g. family history) and proposed to reflect increases in neural compensatory strategies associated with AD neurodegeneration (Bondi et al., 2005; Bookheimer et al., 2000; Green et al., 2023)**.** The presence of this cluster independent of the HV measure used, could be related to the strength of the relationship between hippocampal atrophy and hyperactivation of the precuneus and cuneus.

The significant inverse relationship between Avg-HOC and activity in right and left olfactory cortical regions during correct OID responses suggests heavy reliance on hippocampal integrity. The two clusters were large, though largest in the right hemisphere, and included regions centrally involved in olfactory-stimulus processing (posterior OFC complex, piriform cortex, hippocampus, amygdala), and in early AD tau pathology. CSF t-tau/Aβ_1–42_ levels were negatively associated with the right hemisphere cluster. Increased tau pathology is known to correlate with increased neurodegeneration (i.e., hippocampal atrophy) and cognitive decline (Galasko and Shaw, 2017; Heister et al., 2011), so the observed decrease in right olfactory region BOLD activity may be related to developed tau pathology.

We had found an R^2^ of.91 for the relationship between DRS scores and functional activation in the right parahippocampal gyrus during odor recognition memory in previous research (Kapoulea and Murphy, 2020), and we sought to limit the number of variables in the current analyses, thus right HV and Avg-HOC were analyzed here. Using Avg-HOC as the measurement of HV in Model 2 generally yielded a greater amount of variance explained by the model—indicated by strong adjusted R^2^ values—which supports both the potential value of separately analyzing LHV as well as RHV to probe laterality and future use of Avg-HOC in neurodegeneration and olfactory functioning investigations in pre-clinical AD populations. Larger sample sizes would strengthen future investigations.

## 5. Conclusions

The present use of multivariate linear regression to investigate the complex relationships between AD biomarkers and the neural activity of regions associated with cognitive and olfactory decline in older adults without dementia enabled a greater understanding than univariate biomarker investigations; and use of BOLD thresholds as the dependent variable of the models resulted in insight to neurofunctional differences that may be related to developing AD in older adults without dementia. These findings are limited by sample size but should motivate further investigation of olfactory dysfunction and AD risk factors in older adults without dementia. The specific vulnerability of essential olfactory processing regions in the early stages of AD, as well as their structural and functional associations with AD risk factors and olfactory dysfunction, suggests that combining measures of olfactory dysfunction with other biomarkers may be more indicative of disease progression in older adults without dementia than standard clinical measures alone. Understanding manifestations of underlying AD pathology in cognitively normal adults at risk for the disease would significantly benefit the ability to define and postulate specific treatments for AD.

## Supplementary Material

1

## Figures and Tables

**Table 1:** Regions Showing Significant BOLD Activation during Correct Odor Identification.

Region	Hemisphere	X	Y	Z	T	Cluster Size
Postcentral Gyrus	L	−25	−31	58	10.3	22358
Precentral Gyrus	L	−25	−10	53	10.2	
Superior Medial Gyrus	L	−8	20	40	7.43	
Precentral Gyrus	R	41	−12	57	7.28	
Paracentral Lobule	L	−9	−17	52	7.01	
Rolandic Operculum	R	52	8	1	6.76	
Superior Frontal Gyrus	L	−13	22	43	6.72	
Inferior Parietal Lobule	L	−53	−31	45	6.68	
Fusiform Gyrus	L	−35	−25	−27	6.68	
Cerebellum (VI)	R	27	−58	−26	6.66	
Insula	L	−35	17	−1	6.63	
Middle Cingulate Cortex	R	2	−8	51	6.62	
Inferior Frontal Gyrus (p. Triangularis)	L	−45	34	13	6.31	
Putamen	R	30	−16	3	6.22	
Putamen	L	−29	−16	4	6.08	
Perirhinal Cortex	L	−40	−18	−29	6.05	
Thalamus	L	−13	−16	−2	6.02	
Piriform Cortex	L	−23	−3	−17	5.98	
Parahippocampal Gyrus	R	25	−20	−24	5.76	
Anterior Cingulate Cortex	R	5	26	28	5.72	
Thalamus	R	15	−16	−2	5.67	
Middle Cingulate Cortex	L	−6	−16	45	5.64	
Middle Occipital Gyrus	L	−48	−73	−2	5.6	
Supplementary Motor Area	L	−3	2	53	5.55	
Inferior Frontal Gyrus (p. Orbitalis)	L	−47	41	−4	5.52	
Cerebellum (IV-V)	R	10	−53	−22	5.48	
Postcentral Gyrus	R	42	−31	44	5.43	
Middle Frontal Gyrus	R	30	0	54	5.41	
Middle Frontal Gyrus	L	−34	2	54	5.41	
Cerebellum (VI)	L	−30	−58	−26	5.3	
Entorhinal Cortex	L	−30	−18	−29	5.22	
Cerebellar Vermis (4/5)	​	7	−56	−24	5.18	
Piriform Cortex	R	27	−3	−16	5.11	
Superior Temporal Gyrus	R	−55	−11	5	5.08	
Inferior Frontal Gyrus (p. Opercularis)	R	56	8	16	5.08	
Supramarginal Gyrus	R	60	−17	29	5.03	
Insula	R	43	2	9	5	
Superior Parietal Lobule	L	−25	−67	54	4.91	
Entorhinal Cortex	R	27	−14	−30	4.81	
Precuneus	R	10	−58	64	4.7	
Perirhinal Cortex	R	36	−14	−36	4.64	
Superior Temporal Gyrus	L	−60	−16	8	4.59	
Supplementary Motor Area	R	10	8	53	4.56	
Rolandic Operculum	L	−52	6	4	4.53	
Inferior Frontal Gyrus (p. Triangularis)	R	46	40	9	4.38	
Lingual Gyrus	R	7	−89	−10	4.29	
Cerebellum (IV-V)	L	−7	−55	−14	4.24	
Inferior Frontal Gyrus (p. Orbitalis)	R	46	40	−7	4.2	
Precuneus	L	−11	−68	60	4.18	
Inferior Frontal Gyrus (p. Opercularis)	L	−47	13	12	4.12	
Superior Occipital Gyrus	R	28	−82	45	4.03	
Superior Frontal Gyrus	R	26	21	47	3.98	
Anterior Cingulate Cortex	L	−2	26	27	3.98	
Inferior Occipital Gyrus	L	−27	−96	−12	3.97	
Hippocampus	R	27	−9	−19	3.96	
Supramarginal Gyrus	L	−53	−39	35	3.94	
Fusiform Gyrus	R	24	−35	−19	3.84	
Superior Parietal Lobule	R	39	−51	57	3.8	
Inferior Temporal Gyrus	L	−49	−51	−20	3.8	
Angular Gyrus	R	38	−64	46	3.73	
Calcarine Gyrus	L	−7	−96	−9	3.71	
Inferior Parietal Lobule	R	37	−50	54	3.66	
Parahippocampal Gyrus	L	−29	−20	−25	3.57	
Hippocampus	L	−21	−12	−22	3.55	
Lingual Gyrus	L	−13	−72	1	3.52	
Paracentral Lobule	R	8	−32	58	3.51	
White Matter	L	−28	−52	17	−4.13	117
Lingual Gyrus	R	20	−64	−1	3.87	31
Posterior Cingulate Cortex	L	−4	−31	23	4.01	31

**Table 2:** Regression Coefficients for the Clusters of BOLD Associated with APOE-ε4 Allele Status During Correct Odor Identification Responses. Table 2. The clusters of BOLD activity were negatively associated with APOE-ε4 status during correct OID responses. The table shows the unique beta-weight coefficients and corresponding p-values for each significant model variable, the adjusted R^2^ value of each model, and corresponding model F-statistics.

	Left Superior Frontal Lobe		Right Superior Frontal Lobe		Right Med. Temporal Lobe	
Variable	Model 1	Model 2	Model 1	Model 2	Model 1	Model 2
	(N = 36)	(N = 32)	(N = 36)	(N = 32)	(N = 36)	(N = 32)
	β (*P* value)	β (*P* value)	β (*P* value)	β (*P* value)	β (*P* value)	β (*P* value)
APOE-ε4	−0.22 (<.001)	−0.23 (<.001)	−0.32 (<.001)	−0.33 (<.001)	−0.23 (.003)	−0.22 (.053)
CSF	−0.01 (.746)	−0.02 (.586)	−0.02 (.724)	−0.01 (.782)	−0.07 (.071)	−0.01 (.759)
HV	−40.66 (.443)	−0.28 (.220)	71.66 (.361)	0.21 (.517)	−1.11 (.985)	−0.26 (.220)
Age_75	−0.02 (.006)	−0.02 (.010)	−0.03 (<.001)	−0.03 (.005)	−0.02 (.019)	−0.01 (.141)
Female	−0.10 (.027)	−0.08 (.086)	−0.11 (.089)	−0.10 (.113)	−0.04 (.486)	−0.09 (.048)
ε4xSex	0.21 (.003)	0.19 (.011)	0.28 (.006)	0.25 (.021)	0.20 (.010)	0.18 (.011)
AgexSex Adj	0.02 (.017)	0.02 (.033)	0.043 (.001)	0.05 (.002)	0.02 (.075)	0.01 (.119)
R^2^	0.337	0.382	0.442	0.486	0.369	0.406
F (*P* value)	3.54 (.008)	3.65 (.009)	4.96 (.001)	5.05 (.001)	3.93 (.004)	9.93 (.006)

**Table 3:** Regression Coefficients for the Clusters of BOLD Associated with Hippocampal Volume During Correct Odor Identification Responses. Table 3. Both clusters of BOLD activity were negatively associated with the measure of HV during correct OID responses. The left side of the table shows the statistics from Models 1 & 2 for the cluster of precuneus and cuneus BOLD activity associated with both Right HV/ICV and Avg-HOC; the right side shows the statistics for the two additional clusters found, only by Model 2, to relate to Avg-HOC during correct OID. The table shows the unique beta-weight coefficients and corresponding p-values for each significant model variable, the adjusted R^2^ value of each model, and corresponding model F-statistics.

	Precuneus & Cuneus		Right Olf	Left Olf
Variable	Model 1	Model 2	Model 2	Model 2
	(N = 36)	(N = 32)	(N = 32)	(N = 32)
	β (*P* value)	β (*P* value)	β (*P* value)	β (*P* value)
APOE-ε4	0.55 (.183)	−0.05 (.403)	−0.05 (.394)	0.06 (.743)
CSF	0.44 (.641)	−0.06 (.112)	−0.09 (.025)	−0.07 (.497)
HV	−38.12 (<.001)	−1.13 (<.001)	−1.20 (<.001)	−3.01 (<.001)
Age_75	−0.39 (.530)	−0.02 (.006)	−0.03 (<.001)	−0.04 (.106)
Female	0.62 (.096)	−0.05 (.359)	−0.05 (.355)	0.30 (.060)
ε4xSex	−0.47 (.372)	0.005 (.955)	−0.04 (.615)	−0.14 (.574)
AgexSex Adj	0.22 (.352)	0.02 (.079)	0.03 (.009)	0.01 (.735)
R^2^	0.299	0.365	0.470	0.261
F (*P* value)	3.13 (.014)	3.47 (.011)	4.80 (.002)	2.52 (.045)

**Table 4:** Regression Coefficients for the Clusters of BOLD Associated with APOE-ε4 Allele Status During Incorrect Odor Identification Responses. Table 4. The clusters of BOLD activity were negatively associated with APOE-ε4 status during incorrect OID responses. The table shows the unique beta-weight coefficients and corresponding *P*-values for each significant model variable, the adjusted R^2^ value of each model, and corresponding model F-statistics.

	Left Corpus Striatum		Left Mid Occipital Gyrus		Left MTL	
Variable	Model 1	Model 2	Model 1	Model 2	Model 1	Model 2
	(N = 36)	(N = 32)	(N = 36)	(N = 32)	(N = 36)	(N = 32)
	β (*P* value)	β (*P* value)	β (*P* value)	β (*P* value)	β (*P* value)	β (*P* value)
APOE-ε4	−0.24 (<.001)	−0.25 (.047)	−0.22 (<.001)	−0.26 (<.001)	−0.38 (<.001)	−0.41 (<.001)
CSF	−0.11 (.009)	0.02 (.467)	0.01 (.662)	−0.11 (.010)	−0.06 (.377)	−0.05 (.386)
HV	−2.82 (.966)	0.31 (.189)	3.85 (.946)	−0.30 (.308)	26.94 (.795)	−0.29 (.487)
Age_75	−0.02 (.016)	−0.004 (.488)	−0.01 (.129)	−0.02 (.010)	−0.04 (.002)	−0.04 (.003)
Sex	−0.11 (.059)	0.01 (.866)	0.02 (.745)	−0.12 (.044)	−0.20 (.027)	−0.15 (.073)
ε4xSex	0.23 (.008)	0.10 (.191)	0.16 (.031)	0.20 (.032)	0.39 (.005)	0.37 (.009)
AgexSex Adj R^2^	0.02 (.076)	0.01 (.444)	−0.001 (.942)	0.03 (.012)	0.03 (.036)	0.04 (.030)
	0.366	0.516	0.390	0.454	0.307	0.409
F (*P* value)	3.89 (.004)	5.57 (<.001)	4.20 (.003)	4.57 (.003)	3.21 (.013)	3.96 (.006)

**Table 5:** Regression Coefficients for the Clusters of BOLD Associated with CSF t-tau/Aβ_1–42_ Levels During Incorrect Odor Identification Responses. Table 5. The clusters of BOLD activity were negatively associated with CSF t-tau/Aβ_1–42_ levels status during incorrect OID responses. The table shows the unique beta-weight coefficients and corresponding p-values for each significant model variable, the adjusted R^2^ value of each model, and corresponding model F-statistics.

	Left OFC & Temporal Pole	
Variable	Model 1 (N = 36)β (*P* value)	Model 2 (N = 32)β (*P* value)
APOE-ε4	−0.18 (.009)	−0.16 (.029)
CSF	−0.21 (.010)	−0.20 (.043)
HV	33.42 (.622)	−0.15 (.629)
Age_75	−0.03 (<.001)	−0.03 (.002)
Sex	−0.18 (.003)	−0.16 (.012)
ε4xFem	0.19 (.027)	0.14 (.158)
AgexSex	0.04 (.001)	0.04 (.008)
Adj R^2^	0.553	0.533
F (*P* value)	7.17 (<.001)	5.89 (<.001)

## Appendix A. Supporting information

Supplementary data associated with this article can be found in the online version at doi:10.1016/j.neurobiolaging.2025.02.001.

## Abbreviations:

Avg-HOC: average hippocampal occupancy
EC: entorhinal cortex
HV: hippocampal volume
ICV: estimated intracranial volume
MCC: middle cingulate cortex
MTL: medial temporal lobe
OFC: orbitofrontal cortex
SFG: superior frontal gyrus
SFL: superior frontal lobe
