# Supplementary material for: Brain activation in older adults during odor identification is related to ApoE, t-tau/Aβ1–42, and hippocampal volume
Source: Neurobiol Aging. Author manuscript; Available in PMC 2026 Jul 8. (PMC13345118; doi:10.1016/j.neurobiolaging.2025.02.001)
Supplement: 1 [file NIHMS2171270-supplement-1.docx]

Supplementary Table 1

Patient demographics

Demographics N N_Female_ M_Age_(SD)

Full Sample 36 20 76.61 (4.21)

APOE ε4+ 16 10 76.38 (5.21)

APOE ε4- 20 10 76.80 (3.35)
